# Supplementary material for: CD34+ Hematopoietic Stem Cell Counts in Alzheimer’s Disease: A Meta-Analysis
Source: Diseases. 2025 Jan 21;13(2):25. doi: 10.3390/diseases13020025 (PMC11854744; doi:10.3390/diseases13020025)
Supplement: Supplementary file 1 [file diseases-13-00025-s001.zip › diseases-3403336-supplementary.pdf]

Table S1: Search Query

Database	Studies Retrieved	Search Query
Pubmed	348	1. (((((((((((((((((((((((((((((((((((Alzheimer Syndrome) OR (Alzheimer-Type Dementia (ATD))) OR (Alzheimer Type Dementia (ATD))) OR (Dementia, Alzheimer-Type (ATD))) OR (Alzheimer's Diseases)) OR (Alzheimer Diseases)) OR (Alzheimers Diseases)) OR (Alzheimer Dementia)) OR (Alzheimer Dementias)) OR (Dementia, Alzheimer)) OR (Alzheimer's Disease)) OR (Dementia, Senile)) OR (Senile Dementia)) OR (Dementia, Alzheimer Type)) OR (Alzheimer Type Dementia)) OR (Senile Dementia, Alzheimer Type)) OR (Alzheimer Type Senile Dementia)) OR (Primary Senile Degenerative Dementia)) OR (Alzheimer Sclerosis)) OR (Sclerosis, Alzheimer)) OR (Dementia, Primary Senile Degenerative)) OR (Dementia, Presenile)) OR (Presenile Dementia)) OR (Acute Confusional Senile Dementia)) OR (Senile Dementia, Acute Confusional)) OR (Alzheimer Disease, Early Onset)) OR (Early Onset Alzheimer Disease)) OR (Presenile Alzheimer Dementia)) OR (Alzheimer Disease, Late Onset)) OR (Late Onset Alzheimer Disease)) OR (Alzheimer's Disease, Focal Onset)) OR (Focal Onset Alzheimer's Disease)) OR (Familial Alzheimer Disease (FAD))) OR (Alzheimer Disease, Familial (FAD))) OR (Familial Alzheimer Diseases (FAD))) OR ("Alzheimer Disease"[Mesh])<br><br>2. (((((((((Antigen CD34) OR (CD34 Antigen)) OR (Antigen, CD34)) OR (CD34 Antigens)) OR ("CD34+")) OR (CD34 Positive)) OR (CD34 Expression)) OR (CD 34 Marker)) OR (CD 34 Progenitor Cell)) OR (Progenitor cell, CD34)<br><br>3. ((((((((((((((((((((((((((((((((AC133-1 Antigen) OR (Antigen, AC133)) OR (AC133 1 Antigen)) OR (Antigen, AC133-1)) OR (CD133 Antigen)) OR (Antigen, CD133)) OR (Fudenine)) OR (Prominin-1)) OR (Prominin)) OR (Prominin 1)) OR (Prominin-Like PROML1)) OR (Prominin Like PROML1)) OR (PROML1)) OR (AC141 Antigen)) OR (Antigen, AC141)) OR (AC133-2 Antigen)) OR (AC133 2 Antigen)) OR (Antigen, AC133-2)) OR (AC133 Marker)) OR (AC133 Stem Cells)) OR (AC133 Positive)) OR (AC133 Expression)) OR (AC133 Protein)) OR (AC133 Surface)) OR (Antigen AC133 Immunostaining)) OR (AC133+ Cells)) OR (AC133 Antibody)) OR (AC133+ Hematopoietic Progenitor))) OR (AC133+ Stem Cell Isolation)) OR (AC133+ Progenitor Cells)) OR (Cells AC133 Endothelial Cells)) OR (AC133+ Hematopoietic Stem Cells)<br><br>4. ((((((((((((CD Antigens) OR (CD Antigen)) OR (Antigen, CD)) OR (Cluster of Differentiation Antigen)) OR (Antigen Cluster, Differentiation)) OR (Differentiation Antigen Cluster)) OR (Cluster of Differentiation Marker)) OR (Differentiation Marker Cluster)) OR (Marker Cluster, Differentiation)) OR (Cluster of Differentiation Antigens)) OR (Cluster of Differentiation Markers)) OR (Leukocyte Differentiation Antigens, Human)) OR (Differentiation Antigens, Leukocyte, Human)<br><br>5. #1 AND (#2 OR #3 OR #4)
Embase	5	1. 'Alzheimer Syndrome':ti,ab OR 'Alzheimer-Type Dementia (ATD)':ti,ab OR 'Alzheimer Type Dementia (ATD)':ti,ab OR 'Dementia, Alzheimer-Type (ATD)':ti,ab OR 'Alzheimer's Diseases':ti,ab OR 'Alzheimer Diseases':ti,ab OR 'Alzheimers Diseases':ti,ab OR 'Alzheimer Dementia':ti,ab OR 'Alzheimer Dementias':ti,ab OR 'Dementia, Alzheimer':ti,ab OR 'Alzheimer's Disease':ti,ab OR 'Dementia, Senile':ti,ab OR 'Senile Dementia':ti,ab OR 'Dementia, Alzheimer Type':ti,ab OR 'Alzheimer Type Dementia':ti,ab OR 'Senile Dementia, Alzheimer Type':ti,ab OR 'Alzheimer Type Senile Dementia':ti,ab OR 'Primary Senile Degenerative Dementia':ti,ab OR 'Alzheimer Sclerosis':ti,ab OR 'Sclerosis, Alzheimer':ti,ab OR 'Dementia, Primary Senile Degenerative':ti,ab OR 'Dementia, Presenile':ti,ab OR 'Presenile Dementia':ti,ab OR 'Acute Confusional Senile Dementia':ti,ab OR 'Senile Dementia, Acute Confusional':ti,ab OR 'Alzheimer Disease, Early Onset':ti,ab

		OR 'Early Onset Alzheimer Disease':ti,ab OR 'Presenile Alzheimer Dementia':ti,ab OR 'Alzheimer Disease, Late Onset':ti,ab OR 'Late Onset Alzheimer Disease':ti,ab OR 'Alzheimer's Disease, Focal Onset':ti,ab OR 'Focal Onset Alzheimer's Disease':ti,ab OR 'Familial Alzheimer Disease (FAD)':ti,ab OR 'Alzheimer Disease, Familial (FAD)':ti,ab OR 'Familial Alzheimer Diseases (FAD)':ti,ab OR 'Alzheimer Disease':ti,ab<br><br>2. 'CD34 Antigen':ti,ab OR 'Antigen, CD34':ti,ab OR 'CD34 Antigens':ti,ab OR 'CD34+':ti,ab OR 'CD34 Positive':ti,ab OR 'CD34 Expression':ti,ab OR 'CD 34 Marker':ti,ab<br><br>3. 'Antigen, AC133':ti,ab OR 'AC133-1 Antigen':ti,ab OR 'AC133 1 Antigen':ti,ab OR 'Antigen, AC133-1':ti,ab OR 'CD133 Antigen':ti,ab OR 'Antigen, CD133':ti,ab OR 'Fudenine':ti,ab OR 'Prominin':ti,ab OR 'Prominin-1':ti,ab OR 'Prominin 1':ti,ab OR 'Prominin-Like PROML1':ti,ab OR 'Prominin Like PROML1':ti,ab OR 'PROML1':ti,ab OR 'AC141 Antigen':ti,ab OR 'Antigen, AC141':ti,ab OR 'AC133-2 Antigen':ti,ab OR 'AC133 2 Antigen':ti,ab OR 'Antigen, AC133-2':ti,ab OR 'AC133 Marker':ti,ab OR 'AC133 Stem Cells':ti,ab OR 'AC133 Positive':ti,ab OR 'AC133 Expression':ti,ab OR 'AC133 Protein':ti,ab OR 'AC133 Surface':ti,ab OR 'AntigenAC133 Immunostaining':ti,ab OR 'AC133+ Cells':ti,ab OR 'AC133 Antibody':ti,ab OR 'AC133+ Hematopoietic Progenitor':ti,ab OR 'CellsAC133 Endothelial Cells':ti,ab OR 'AC133+ Progenitor Cells':ti,ab OR 'AC133+ Stem Cell Isolation':ti,ab OR 'AC133+ Hematopoietic Stem Cells':ti,ab<br><br>4. 'CD Antigens':ti,ab OR 'CD Antigen':ti,ab OR 'Antigen, CD':ti,ab OR 'Cluster of Differentiation Antigen':ti,ab OR 'Antigen Cluster, Differentiation':ti,ab OR 'Differentiation Antigen Cluster':ti,ab OR 'Cluster of Differentiation Marker':ti,ab OR 'Differentiation Marker Cluster':ti,ab OR 'Marker Cluster, Differentiation':ti,ab OR 'Cluster of Differentiation Antigens':ti,ab OR 'Cluster of Differentiation Markers':ti,ab OR 'Leukocyte Differentiation Antigens, Human':ti,ab OR 'Differentiation Antigens, Leukocyte, Human':ti,ab<br><br>5. #2 0R #3 OR #4<br><br>6. #1 AND #5
Cochrane	2	#1 Alzheimer Syndrome OR Alzheimer-Type Dementia (ATD) OR Alzheimer Type Dementia (ATD) OR Dementia, Alzheimer-Type (ATD) OR Alzheimer's Diseases OR Alzheimer Diseases OR Alzheimers Diseases OR Alzheimer Dementia OR Alzheimer Dementias OR Dementia, Alzheimer OR Alzheimer's Disease OR Dementia, Senile OR Senile Dementia OR Dementia, Alzheimer Type OR Alzheimer Type Dementia OR Senile Dementia, Alzheimer Type OR Alzheimer Type Senile Dementia OR Primary Senile Degenerative Dementia OR Alzheimer Sclerosis OR Sclerosis, Alzheimer OR Dementia, Primary Senile Degenerative OR Dementia, Presenile OR Presenile Dementia OR Acute Confusional Senile Dementia OR Senile Dementia, Acute Confusional OR Alzheimer Disease, Early Onset OR Early Onset Alzheimer Disease OR Presenile Alzheimer Dementia OR Alzheimer Disease, Late Onset OR Late Onset Alzheimer Disease OR Alzheimer's Disease, Focal Onset OR Focal Onset Alzheimer's Disease OR Familial Alzheimer Disease (FAD) OR Alzheimer Disease, Familial (FAD) OR Familial Alzheimer Diseases (FAD) OR "Alzheimer Disease"<br><br>#2 CD34 Antigen OR Antigen, CD34 OR CD34 Antigens OR “CD34” OR CD34 Positive OR CD34 Expression OR CD 34 Marker<br><br>#3 Antigen, AC133 OR AC133 1 Antigen OR AC133 1 Antigen OR Antigen, AC133 1 OR CD133 Antigen OR Antigen, CD133 OR Fudenine OR Prominin OR Prominin 1 OR Prominin 1 OR Prominin Like PROML1 OR Prominin Like PROML1 OR PROML1 OR AC141 Antigen OR Antigen, AC141 OR AC133 2 Antigen OR AC133 2 Antigen OR Antigen, AC133 2 OR AC133 Marker OR AC133 Stem Cells OR AC133 Positive OR AC133 Expression OR AC133 Protein OR AC133 Surface OR AntigenAC133 Immunostaining OR AC133 Cells OR AC133 Antibody OR AC133 Hematopoietic Progenitor Cells OR AC133 Endothelial Cells OR AC133 Progenitor Cells OR AC133 Stem Cell Isolation OR AC133 Hematopoietic Stem Cells

		#4 CD Antigens OR CD Antigen OR Antigen, CD OR Cluster of Differentiation Antigen OR Antigen Cluster, Differentiation OR Differentiation Antigen Cluster OR Cluster of Differentiation Marker OR Differentiation Marker Cluster OR Marker Cluster, Differentiation OR Cluster of Differentiation Antigens OR Cluster of Differentiation Markers OR Leukocyte Differentiation Antigens, Human OR Differentiation Antigens, Leukocyte, Human<br><br>#5 #1 AND (#2 OR #3 OR #4)
Scopus	319	(TITLE-ABS-AKEY("Alzheimer Syndrome") OR TITLE-ABS-KEY("Alzheimer-Type Dementia (ATD)") OR TITLE-ABS-KEY("Alzheimer Type Dementia (ATD)") OR TITLE-ABS-KEY("Dementia, Alzheimer-Type (ATD)") OR TITLE-ABS-KEY("Alzheimer"s Diseases") OR TITLE-ABS-KEY("Alzheimer Diseases") OR TITLE-ABS-KEY("Alzheimers Diseases") OR TITLE-ABS-KEY("Alzheimer Dementia") OR TITLE-ABS-KEY("Alzheimer Dementias") OR TITLE-ABS-KEY("Dementia, Alzheimer") OR TITLE-ABS-KEY("Alzheimer"s Disease") OR TITLE-ABS-KEY("Dementia, Senile") OR TITLE-ABS-KEY("Senile Dementia") OR TITLE-ABS-KEY("Dementia, Alzheimer Type") OR TITLE-ABS-KEY("Alzheimer Type Dementia") OR TITLE-ABS-KEY("Senile Dementia, Alzheimer Type") OR TITLE-ABS-KEY("Alzheimer Type Senile Dementia") OR TITLE-ABS-KEY("Primary Senile Degenerative Dementia") OR TITLE-ABS-KEY("Alzheimer Sclerosis") OR TITLE-ABS-KEY("Sclerosis, Alzheimer") OR TITLE-ABS-KEY("Dementia, Primary Senile Degenerative") OR TITLE-ABS-KEY("Dementia, Presenile") OR TITLE-ABS-KEY("Presenile Dementia") OR TITLE-ABS-KEY("Acute Confusional Senile Dementia") OR TITLE-ABS-KEY("Senile Dementia, Acute Confusional") OR TITLE-ABS-KEY("Alzheimer Disease, Early Onset") OR TITLE-ABS-KEY("Early Onset Alzheimer Disease") OR TITLE-ABS-KEY("Presenile Alzheimer Dementia") OR TITLE-ABS-KEY("Alzheimer Disease, Late Onset") OR TITLE-ABS-KEY("Late Onset Alzheimer Disease") OR TITLE-ABS-KEY("Alzheimer"s Disease, Focal Onset") OR TITLE-ABS-KEY("Focal Onset Alzheimer"s Disease") OR TITLE-ABS-KEY("Familial Alzheimer Disease (FAD)") OR TITLE-ABS-KEY("Alzheimer Disease, Familial (FAD)") OR TITLE-ABS-KEY("Familial Alzheimer Diseases (FAD)") OR TITLE-ABS-KEY("Alzheimer Disease")) AND (TITLE-ABS-KEY("CD34 Antigen") OR TITLE-ABS-KEY("Antigen, CD34") OR TITLE-ABS-KEY("CD34 Antigens") OR TITLE-ABS-KEY("CD34+") OR TITLE-ABS-KEY("CD34 Positive") OR TITLE-ABS-KEY("CD34 Expression") OR TITLE-ABS-KEY("CD 34 Marker") OR TITLE-ABS-KEY("Antigen, AC133") OR TITLE-ABS-KEY("AC133-1 Antigen") OR TITLE-ABS-KEY("AC133 1 Antigen") OR TITLE-ABS-KEY("Antigen, AC133-1") OR TITLE-ABS-KEY("CD133 Antigen") OR TITLE-ABS-KEY("Antigen, CD133") OR TITLE-ABS-KEY("Fudenine") OR TITLE-ABS-KEY("Prominin") OR TITLE-ABS-KEY("Prominin-1") OR TITLE-ABS-KEY("Prominin 1") OR TITLE-ABS-KEY("Prominin-Like PROML1") OR TITLE-ABS-KEY("Prominin Like PROML1") OR TITLE-ABS-KEY("PROML1") OR TITLE-ABS-KEY("AC141 Antigen") OR TITLE-ABS-KEY("Antigen, AC141") OR TITLE-ABS-KEY("AC133-2 Antigen") OR TITLE-ABS-KEY("AC133 2 Antigen") OR TITLE-ABS-KEY("Antigen, AC133-2") OR TITLE-ABS-KEY("AC133 Marker") OR TITLE-ABS-KEY("AC133 Stem Cells") OR TITLE-ABS-KEY("AC133 Positive") OR TITLE-ABS-KEY("AC133 Expression") OR TITLE-ABS-KEY("AC133 Protein") OR TITLE-ABS-KEY("AC133 Surface") OR TITLE-ABS-KEY("AntigenAC133 Immunostaining") OR TITLE-ABS-KEY("AC133+ Cells") OR TITLE-ABS-KEY("AC133 Antibody") OR TITLE-ABS-KEY("AC133+ Hematopoietic Progenitor") OR TITLE-ABS-KEY("CellsAC133 Endothelial Cells") OR TITLE-ABS-KEY("AC133+ Progenitor Cells") OR TITLE-ABS-KEY("AC133+ Stem Cell Isolation") OR TITLE-ABS-KEY("AC133+ Hematopoietic Stem Cells") OR TITLE-ABS-KEY("CD Antigens") OR TITLE-ABS-KEY("CD Antigen") OR TITLE-ABS-KEY("Antigen, CD") OR TITLE-ABS-KEY("Cluster of Differentiation Antigen") OR TITLE-ABS-KEY("Antigen Cluster, Differentiation") OR TITLE-ABS-KEY("Differentiation Antigen Cluster") OR TITLE-ABS-KEY("Cluster of Differentiation Marker") OR TITLE-ABS-KEY("Differentiation Marker Cluster") OR TITLE-ABS-KEY("Marker Cluster, Differentiation") OR TITLE-ABS-KEY("Cluster of Differentiation Antigens") OR TITLE-ABS-KEY("Cluster of Differentiation Markers") OR TITLE-ABS-KEY("Leukocyte Differentiation Antigens, Human") OR TITLE-ABS-KEY("Differentiation Antigens, Leukocyte, Human"))
CTG	59	(Antigen OR CD 34 OR AC133 Antigen OR CD) AND Alzheimer Disease

Table S2: PRISMA Checklist

Section and Topic	Item #	Checklist item (Prevalence of HIV among the Mpox patients: A systematic review and meta-analysis)	Location where item is reported
**TITLE**			
Title	1	Identify the report as a systematic review.	1
**ABSTRACT**			
Abstract	2	See the PRISMA 2020 for Abstracts checklist. (made as per the Journal guidelines)	1
**INTRODUCTION**			
Rationale	3	Describe the rationale for the review in the context of existing knowledge.	1,2
Objectives	4	Provide an explicit statement of the objective(s) or question(s) the review addresses.	1,2
**METHODS**			
Eligibility criteria	5	Specify the inclusion and exclusion criteria for the review and how studies were grouped for the syntheses.	2
Information sources	6	Specify all databases, registers, websites, organisations, reference lists and other sources searched or consulted to identify studies. Specify the date when each source was last searched or consulted.	2
Search strategy	7	Present the full search strategies for all databases, registers and websites, including any filters and limits used.	Table S3
Selection process	8	Specify the methods used to decide whether a study met the inclusion criteria of the review, including how many reviewers screened each record and each report retrieved, whether they worked independently, and if applicable, details of automation tools used in the process.	2
Data collection process	9	Specify the methods used to collect data from reports, including how many reviewers collected data from each report, whether they worked independently, any processes for obtaining or confirming data from study investigators, and if applicable, details of automation tools used in the process.	2
Data items	10a	List and define all outcomes for which data were sought. Specify whether all results that were compatible with each outcome domain in each study were sought (e.g. for all measures, time points, analyses), and if not, the methods used to decide which results to collect.	2,3, Table 1
	10b	List and define all other variables for which data were sought (e.g. participant and intervention characteristics, funding sources). Describe any assumptions made about any missing or unclear information.	2, Table 1
Study risk of bias assessment	11	Specify the methods used to assess risk of bias in the included studies, including details of the tool(s) used, how many reviewers assessed each study and whether they worked independently, and if applicable, details of automation tools used in the process.	2, Table S1, Table S2
Effect measures	12	Specify for each outcome the effect measure(s) (e.g. risk ratio, mean difference) used in the synthesis or presentation of results.	2
Synthesis methods	13a	Describe the processes used to decide which studies were eligible for each synthesis (e.g. tabulating the study intervention characteristics and comparing against the planned groups for each synthesis (item #5)).	2, Table 1
	13b	Describe any methods required to prepare the data for presentation or synthesis, such as handling of missing summary	NA

Section and Topic	Item #	Checklist item (Prevalence of HIV among the Mpox patients: A systematic review and meta-analysis)	Location where item is reported
		statistics, or data conversions.	
	13c	Describe any methods used to tabulate or visually display results of individual studies and syntheses.	Table 1
	13d	Describe any methods used to synthesize results and provide a rationale for the choice(s). If meta-analysis was performed, describe the model(s), method(s) to identify the presence and extent of statistical heterogeneity, and software package(s) used.	2
	13e	Describe any methods used to explore possible causes of heterogeneity among study results (e.g. subgroup analysis, meta-regression).	2
	13f	Describe any sensitivity analyses conducted to assess robustness of the synthesized results.	NA
Reporting bias assessment	14	Describe any methods used to assess risk of bias due to missing results in a synthesis (arising from reporting biases).	NA
Certainty assessment	15	Describe any methods used to assess certainty (or confidence) in the body of evidence for an outcome.	NA
**RESULTS**			
Study selection	16a	Describe the results of the search and selection process, from the number of records identified in the search to the number of studies included in the review, ideally using a flow diagram.	3
	16b	Cite studies that might appear to meet the inclusion criteria, but which were excluded, and explain why they were excluded.	Table 1
Study characteristics	17	Cite each included study and present its characteristics.	Table 1
Risk of bias in studies	18	Present assessments of risk of bias for each included study.	Table S1, Table S2
Results of individual studies	19	For all outcomes, present, for each study: (a) summary statistics for each group (where appropriate) and (b) an effect estimate and its precision (e.g. confidence/credible interval), ideally using structured tables or plots.	3
Results of syntheses	20a	For each synthesis, briefly summarise the characteristics and risk of bias among contributing studies.	3
	20b	Present results of all statistical syntheses conducted. If meta-analysis was done, present for each the summary estimate and its precision (e.g. confidence/credible interval) and measures of statistical heterogeneity. If comparing groups, describe the direction of the effect.	3
	20c	Present results of all investigations of possible causes of heterogeneity among study results.	3
	20d	Present results of all sensitivity analyses conducted to assess the robustness of the synthesized results.	NA
Reporting biases	21	Present assessments of risk of bias due to missing results (arising from reporting biases) for each synthesis assessed.	NA
Certainty of evidence	22	Present assessments of certainty (or confidence) in the body of evidence for each outcome assessed.	NA
**DISCUSSION**			
Discussion	23a	Provide a general interpretation of the results in the context of other evidence.	4

Section and Topic	Item #	Checklist item (Prevalence of HIV among the Mpox patients: A systematic review and meta-analysis)	Location where item is reported
	23b	Discuss any limitations of the evidence included in the review.	4
	23c	Discuss any limitations of the review processes used.	4
	23d	Discuss implications of the results for practice, policy, and future research.	4
**OTHER INFORMATION**			
Registration and protocol	24a	Provide registration information for the review, including register name and registration number, or state that the review was not registered.	NA
	24b	Indicate where the review protocol can be accessed, or state that a protocol was not prepared.	NA
	24c	Describe and explain any amendments to information provided at registration or in the protocol.	NA
Support	25	Describe sources of financial or non-financial support for the review, and the role of the funders or sponsors in the review.	1
Competing interests	26	Declare any competing interests of review authors.	1
Availability of data, code and other materials	27	Report which of the following are publicly available and where they can be found: template data collection forms; data extracted from included studies; data used for all analyses; analytic code; any other materials used in the review.	1

From: Page MJ, McKenzie JE, Bossuyt PM, Boutron I, Hoffmann TC, Mulrow CD, et al. The PRISMA 2020 statement: an updated guideline for reporting systematic reviews. BMJ 2021;372:n71. doi: 10.1136/bmj.n71

Table S3: Risk of Bias Assessment using the ROB 2 Tool for studies included in the Meta-Analysis

Study	D1	D2	D3	D4	D5	D6	D7	D8	Overall
Xiao Dong et al, 2011	*	*		*	**	*	*		Low Risk
Stellos et al, 2010	*	*		*	**	*	*		Low Risk
Bigalke et al, 2011	*	*		*	**	*	*		Low Risk
Maler et al, 2006	*	*	*	*	**	*	*		Low Risk

D1: Selection: Case Definition.

D2: Selection: Case Representativeness.

D3: Selection: Control Selection.

D4: Selection: Control Definition.

D5: Comparability of cases and controls based on study design and analysis.

D6: Exposure: Ascertainment of exposure.

D7: Exposure: Uniform method of ascertainment across case and control.

D8: Exposure: Non-Response Rate.

Table S4: Risk of Bias Assessment using the ROBINS-I Tool for studies included in the Meta-Analysis

Study	D1	D2	D3	D4	D5	D6	D7	Overall
John Ert al, 2012	No Information	Serious Risk	Serious Risk	Moderate Risk	Moderate Risk	Moderate Risk	Serious Risk	Serious Risk

D1: Confounding.

D2: Selection of participants.

D3: Classification of Interventions.

D4: Deviations from intended interventions.

D5: Missing data.

D6: Measurement of outcomes.

D7: Selection of reported results.
